# A case report and literature review of immune checkpoint inhibitor-associated pneumonia caused by penpulimab

**DOI:** 10.3389/fimmu.2023.1114994

**Published:** 2023-06-22

**Authors:** Rongmao Gao, Fuxun Yang, Chen Yang, Zhao Zhang, Mingzong Liu, Chunlin Xiang, Huan Hu, Xiaoxiu Luo, Jiajia Li, Rongan Liu

**Affiliations:** Department of Intensive Care Unit (ICU), Sichuan Provincial People’s Hospital, University of Electronic Science and Technology of China, Chengdu, China

**Keywords:** checkpoint inhibitor pneumonitis, immune checkpoint inhibitor, metagenomic next-generation sequencing, ICU, severe infection

## Abstract

**Objective:**

From the perspective of intensive care physicians, this paper reviews the diagnosis and treatment of CIP patients, analyzes and refines relevant literature on CIP. To summarize the characteristics of diagnosis and treatment of severe CIP provides the basis and reference for early identification, diagnosis and treatment.

**Methods:**

A case of severe CIP caused by piamprilizumab and ICI was reviewed and the literature was reviewed.

**Results:**

This was a patient with lung squamous cell carcinoma with lymphoma who had been treated with multiple chemoradiotherapy and immunotherapy with piamprizumab. The patient was admitted to the ICU with respiratory failure. The intensive care physician performs anti-infective, fluid management, hormonal anti-inflammatory, respiratory and nutritional support treatment, and relies on mNGS to exclude severe infection and CIP treatment, thus successfully saving the patient's life and improving discharge.

**Conclusions:**

The incidence of CIP is very low, and its diagnosis should be combined with clinical manifestations and previous drug use. mNGS can provide certain value in the exclusion of severe infections, so as to provide basis and reference for the early identification, diagnosis and treatment of severe CIP.

## Introduction

Since the time they were first used to treat melanoma in 2011, immune checkpoint inhibitors (ICI) have been widely used for tumor diseases and play an indispensable role as the second-line treatment of all types of tumors because they can enhance immunomodulatory antibodies of the immune system and significantly improve the prognosis of patients with advanced malignant tumors. However, with increased application, immune-related side effects, known as immune-related adverse events, also increase. Penpulimab is a humanized monoclonal antibody against immunoglobulin G1 (IgG1), which binds to programmed cell death protein 1 (PD-1) receptors to block their interaction with programmed death-ligand (PD-L)1 and PD-L2, thereby blocking the immunosuppressive response mediated by the PD-1 pathway, including the anti-tumor immune response (1). Pneumonia is a rare complication of checkpoint inhibitor immunotherapy, with an incidence of approximately 3–5%, but can cause serious consequences and even be fatal when it occurs (2–4). The clinical manifestations of checkpoint inhibitor pneumonitis (CIP) vary; the most common symptoms are dyspnea (53%) and cough (35%), whereas one-third of the patients have no obvious symptoms (5). Chest computed tomography (CT) is the first choice in imaging examinations, although there are no characteristic imaging findings. Routine chest CT reveals no new radiological abnormalities in nearly a quarter of the cases (6). CIP is an exclusive diagnosis, and diseases such as infections and malignant tumors need to be excluded. This is especially crucial for critically ill patients because CIP is easily confused with cytokine release syndrome and is often misdiagnosed as severe pneumonia or acute respiratory distress syndrome (ARDS) (7), thus resulting in the missing of the optimal time window for treatment. Intensive care physicians should understand these kinds of drugs and be familiar with the occurrence and development process of these adverse events to advance the diagnosis and treatment window period of critically ill patients and achieve targeted and accurate treatment, to reduce mortality. From the perspective of intensive care physicians, this paper reviews patient diagnosis and treatment approaches, analyzes and refines the relevant literature on CIP, and summarizes the characteristics of diagnosis and treatment of severe CIP to provide a basis and reference for the early identification, diagnosis, and treatment of patients with severe CIP.

## Case presentation

**Figure 1:**
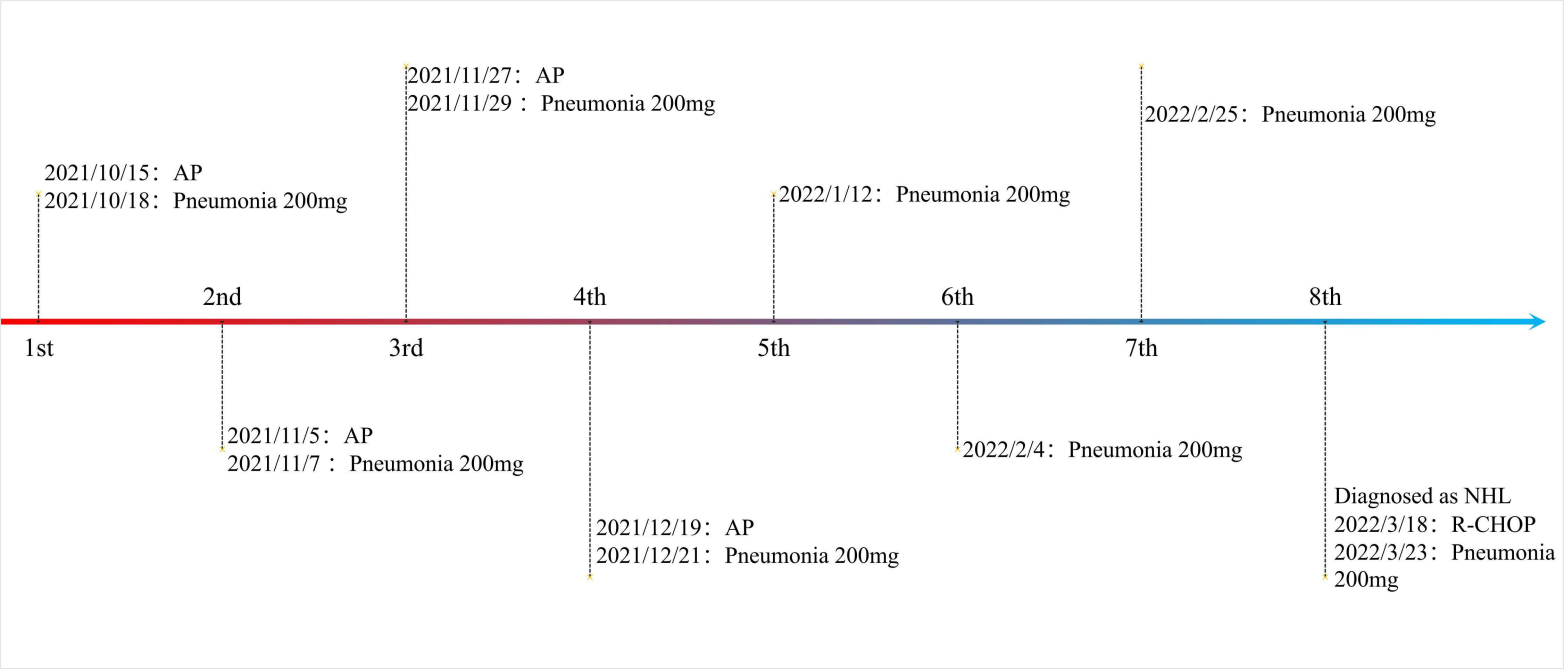
The using of ICI drugs.

**Figure 2:**
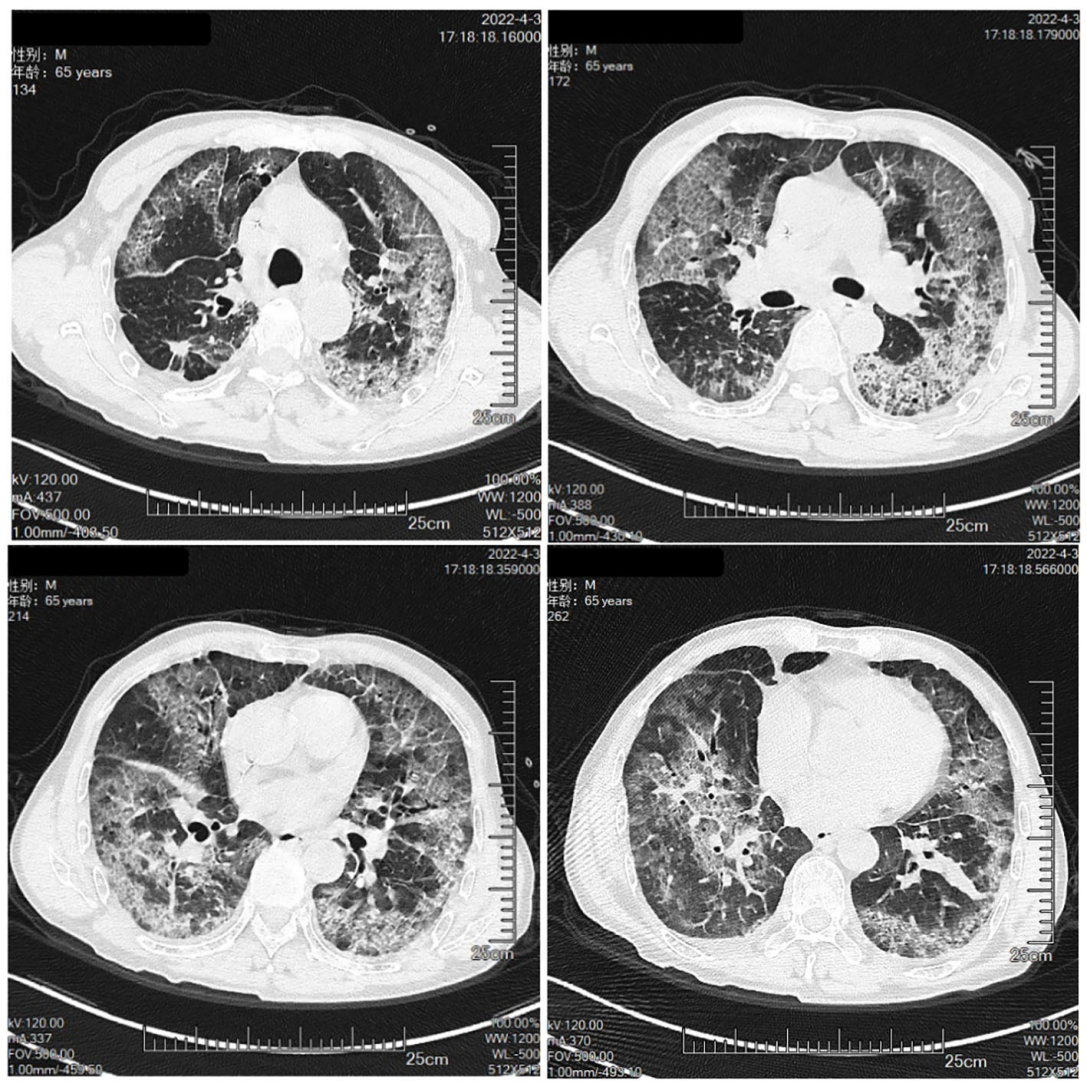
The Chest CT on April 3rd.

**Figure 3:**
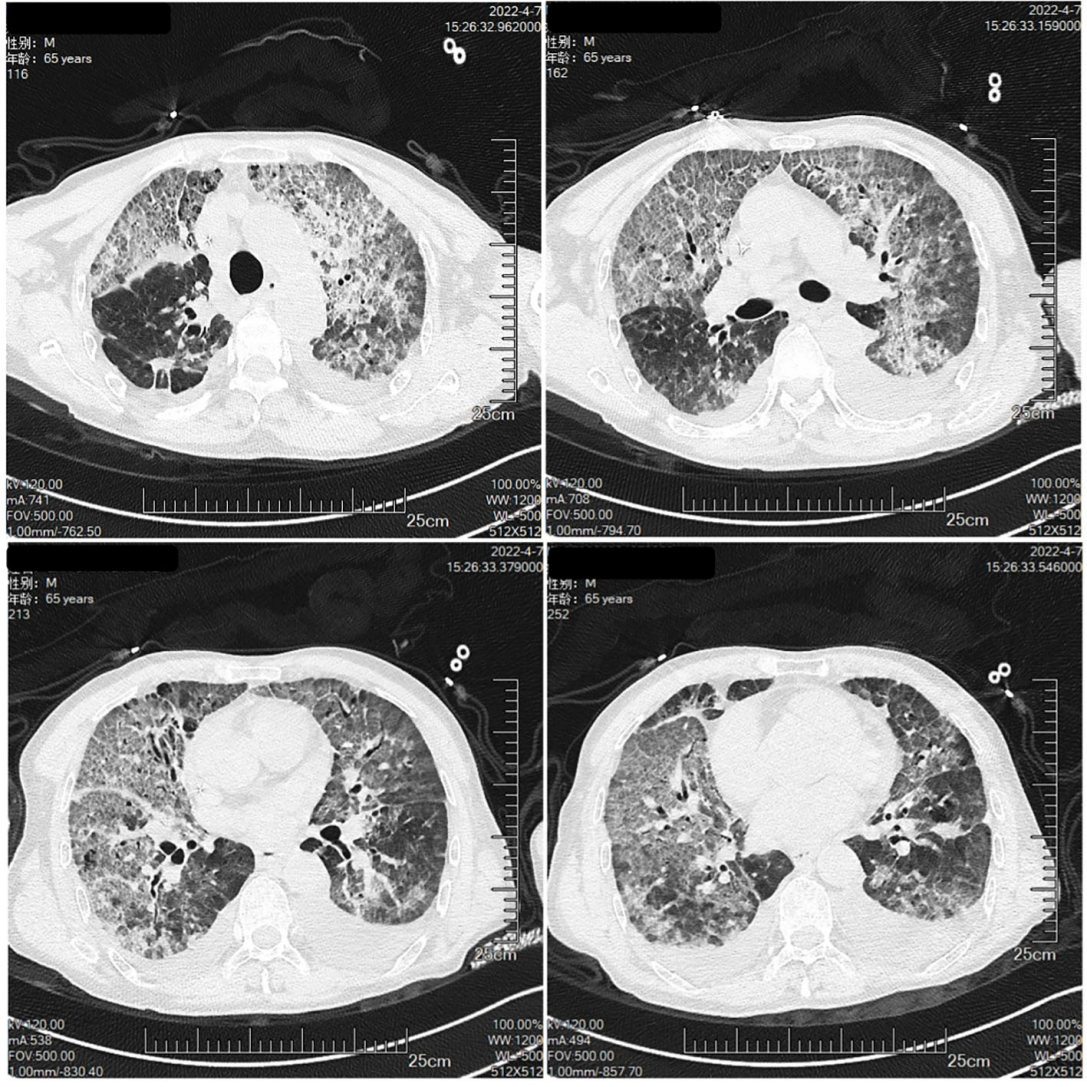
The Chest CT on April 7th.

The patient was a 65-year-old man, with a body mass index of 15.2 Kg/m^2^ and a smoking index of 800 (40 years × 20 cigarettes). In September 2021, the patient was admitted to the hospital for lung occupancy with enlarged lymph nodes in the right neck; a new biopsy of bronchial neoplasm in the lower lobe of the left lung led to the diagnosis of squamous carcinoma with liver metastasis and a biopsy of lymphatic tissue on the right neck as lymphoid hyperplasia with necrosis. The patient received four cycles of albumin paclitaxel combined with lobaplatin regimen chemotherapy and penpulimab immunotherapy from October to December 2021, and the lymph nodes on the right side of the neck shrank significantly. Treatment with cycles 5-7 of penpulimab was continued from January to February 2022. In mid-January 2022, the patient showed enlarged lymph nodes on the left side of the neck, and positron emission tomography-CT showed that the left lower lobe of the lung had increased density in the form of strips and mild fluorodeoxyglucose F 18 uptake. Considering that the tumor activity was suppressed after treatment, the systemic lymph nodes were enlarged, and fluorodeoxyglucose F 18 metabolism was active. A biopsy of the left cervical lymph node ruled out metastatic cancer, and in combination with immunohistochemistry, non-Hodgkin’s lymphoma (NHL) was diagnosed in February 2022. Chemotherapy with the R-CHOP (rituximab, cyclophosphamide, hydroxydaunorubicin hydrochloride (doxorubicin hydrochloride), vincristine (Oncovin), and prednisone) regimen was administered on March 18, 2022, and cycle 8 penpulimab treatment was administered on March 23, 2022 (**Figure 1**). On March 30, 2022, he developed a fever, with the highest body temperature being 39°C, cough, a small amount of white foamy sputum, and dyspnea; cefdinir and ibuprofen proved ineffective. On April 3, the patient was admitted to the Respiratory Department for persistent cough and dyspnea since Mar 30, 2022, and **Figure 2** shows the patient’s CT. The patient’s condition was diagnosed as severe pneumonia type I respiratory failure. Broad-spectrum antibiotics were administered, and the patient was transferred to the intensive care unit on April 9, 2022, for the treatment of worsening dyspnea, increased pulmonary infiltrates (**Figure 3**), and poor oxygenation index.

At the time of admission to the intensive care unit, the patient had severe hypoxemia with an oxygen sum index of 131 and cellular immunodeficiency. Pathogenic test results were negative for galactomannan antigen test, TORCH, nine respiratory pathogens *via* nucleic acid tests, *Pneumocystis* pneumonia, blood cultures, and sputum cultures except for elevated serum (1, 3)-β-D glucan of 199.32 pg/mL. The patient showed no distinct pathogenic evidence of a poor anti-infective effect. It is noteworthy that the patient was on penpulimab a week before the current onset, which is an ICI and has the potential to cause CIP. The patient’s lung imaging showed progressive interstitial pneumonia with persistent fever, poor anti-infective effect, and a high systemic inflammatory response, which could not be explained by infection. Moreover, the use of rituximab in the R-CHOP regimen for lymphoma, where anti-CD20-mediated B-cell depletion has a synergistic effect with PD-1 blockers to promote cytotoxic T-lymphocyte activation, may have increased CIP risk (8, 9). Therefore, the possibility of CIP was considered in the patient. Since CIP requires the exclusion of infection as well as other diseases, the bronchoalveolar lavage fluid was collected and sent for metagenomic next-generation sequencing (mNGS) to exclude infection. Further, the patient tested negative for autoimmune-related antibodies, thus excluding interstitial lung changes due to autoimmune diseases.

**Figure 4:**
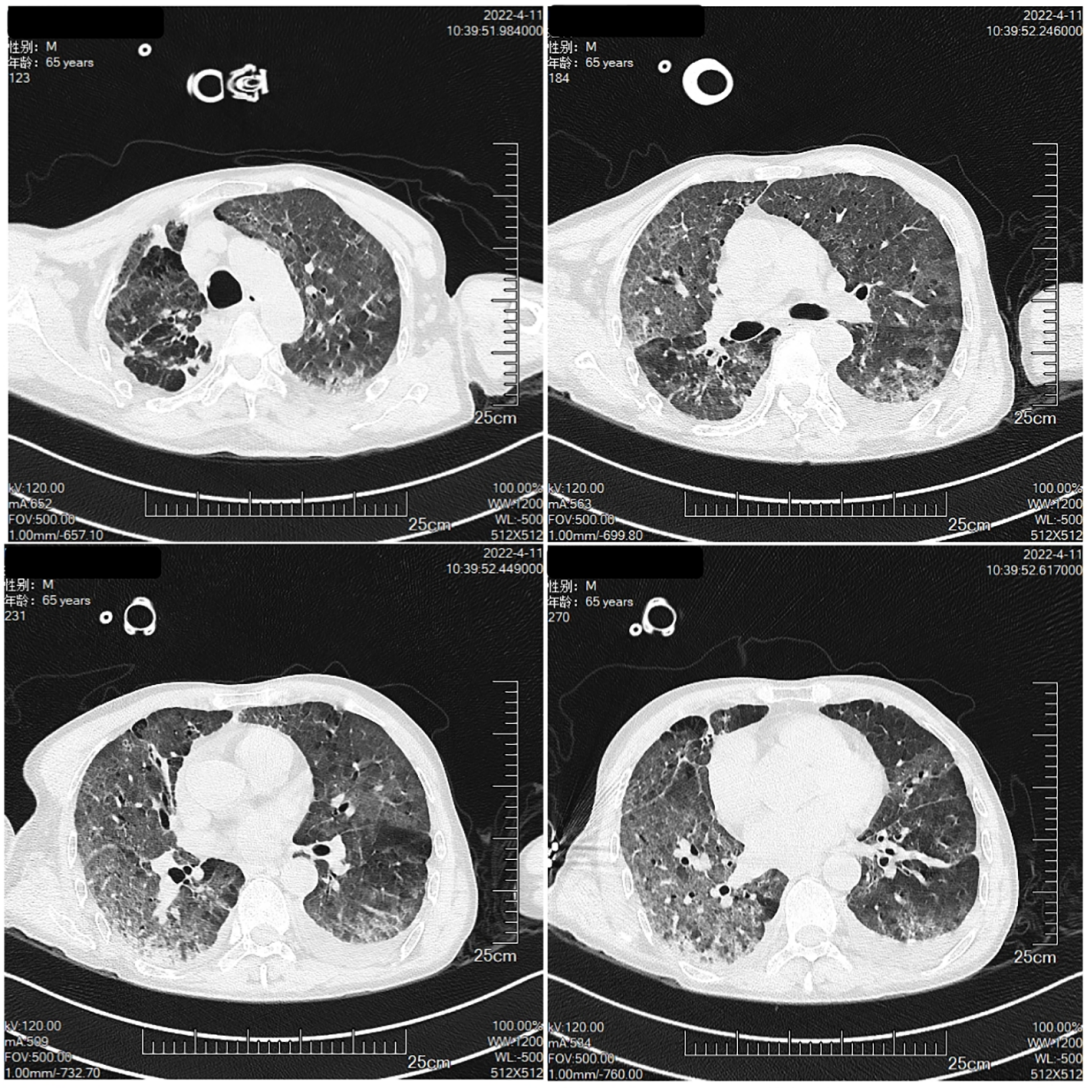
The Chest CT on April 11th.

**Figure 5:**
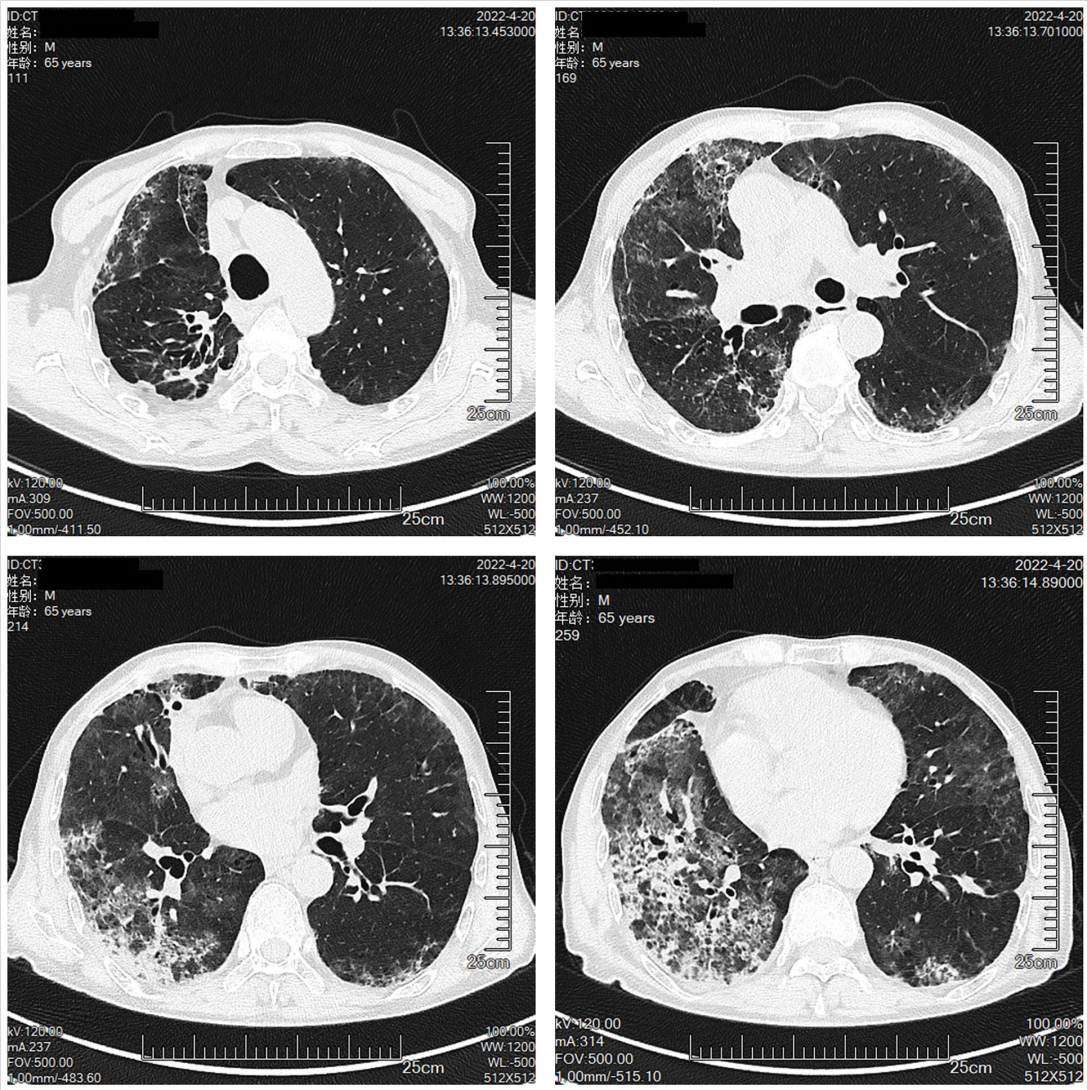
The Chest CT on April 20th.

**Figure 6:**
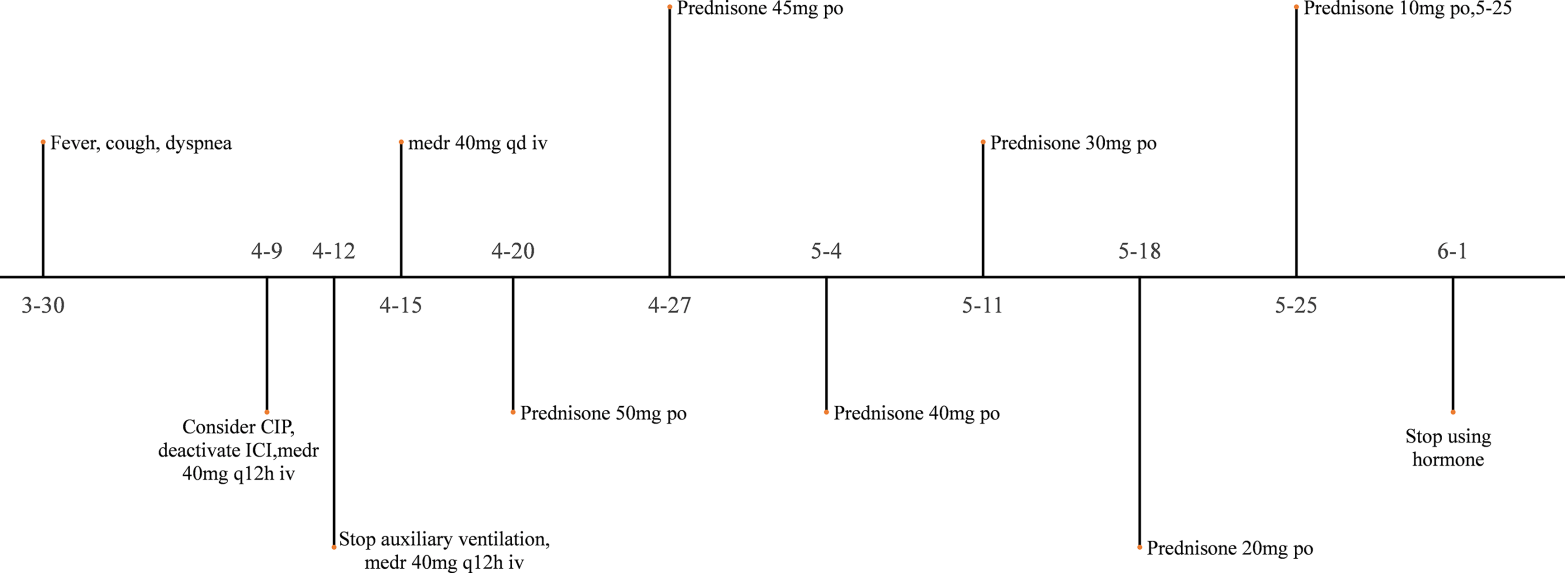
The using of hormone.

**Figure 7:**
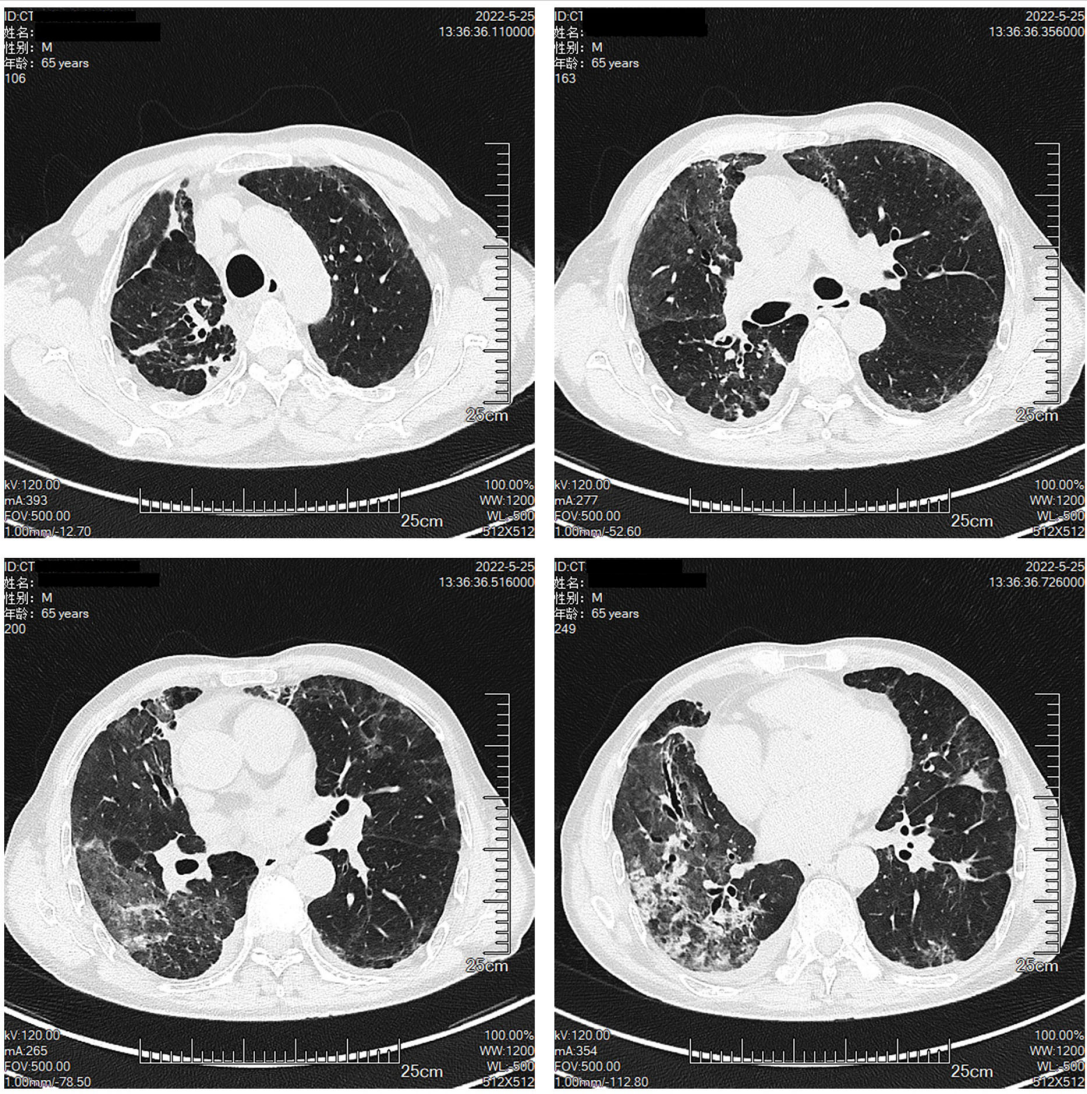
The Chest CT on May 25th.

**Figure 8:**
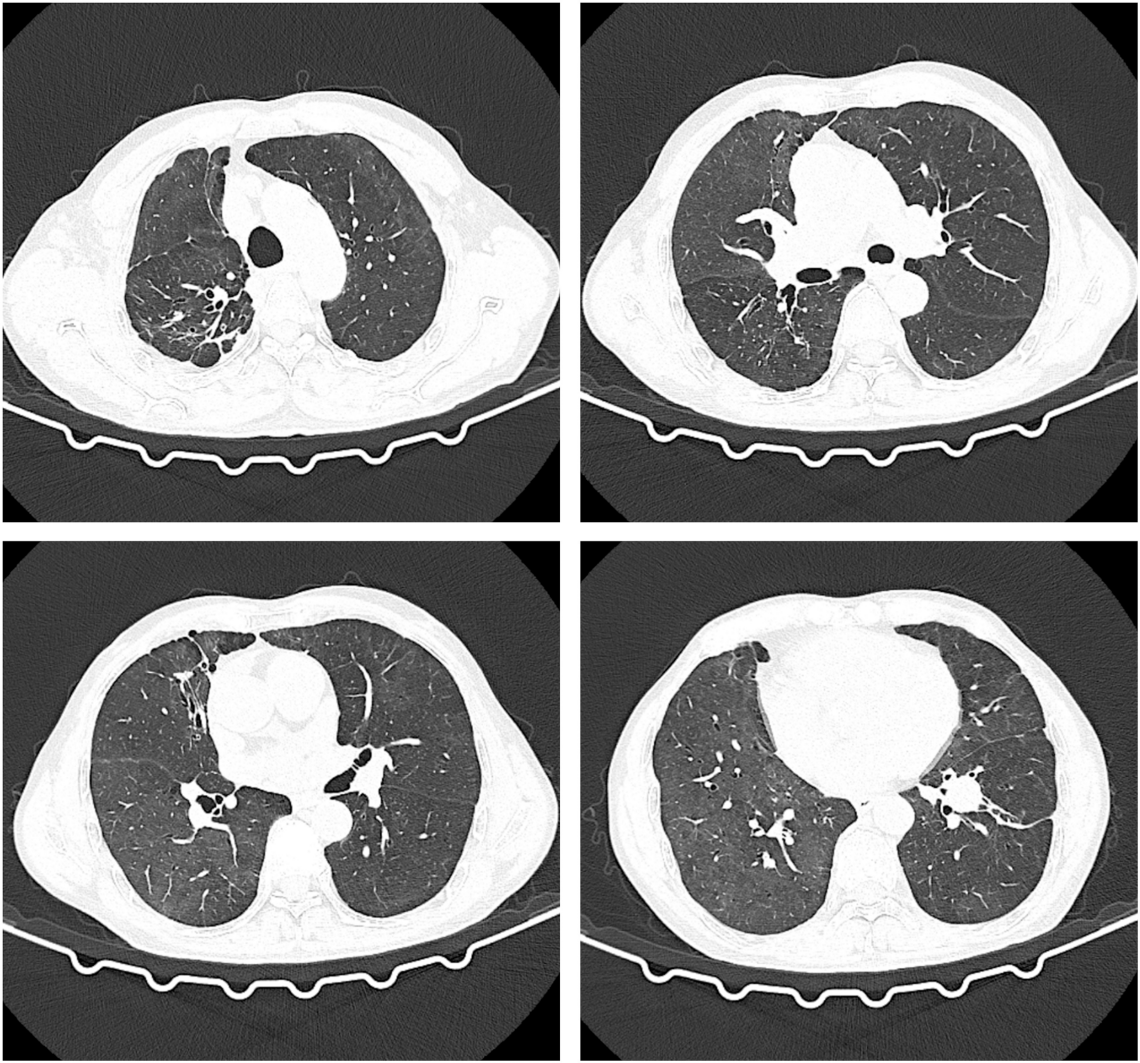
The Chest CT on August 22nd.

The patient had a large lesion in the entire lung and severe respiratory failure. Considering that the patient was diagnosed as having CIP grade 4, methylprednisolone 2 mg/kg (ESMO guideline recommended dose) was administered based on empirical anti-infection (10). The patient had hypoxemia, with an SPO2 of 66% and a respiratory rate of 32 bpm, requiring further respiratory support; therefore, noninvasive mechanical ventilation was chosen as the initial respiratory support strategy. The patient was conscious and had good treatment compliance and airway self-purification; therefore, he was ventilated in the awake prone position during noninvasive ventilation, and his respiratory status was closely monitored during this period. After the above treatment, the patient’s clinical symptoms resolved significantly on April 10, 2022, while the mNGS results showed no pathogenic microorganisms, thus ruling out severe pneumonia and allowing the discontinuation of antibiotics. Chest CT was repeated on April 11, 2022, for bilateral lung lesion absorption (**Figure 4**). The patient was removed from the ventilator and transferred to the respiratory department on April 12, 2022. The methylprednisolone dose was reduced to 40 mg a day on April 15, 2022. Oxygen therapy was discontinued on April 20, 2022, and a repeat CT indicated further absorption of bilateral lung lesions (**Figure 5**), The hormone dose was reduced to prednisone 50 mg, and the patient was discharged with a weekly dose reduction of 5-10 mg (**Figure 6**). As of May 25, 2022, the patient had no restrictions in daily activities, respiratory distress, or other manifestations, and the CT was reviewed in the outpatient clinic (**Figure 7**). At the follow-up on August 22, 2022, the patient underwent a repeat CT as an outpatient, which showed a visible occupying lesion in the left lung (**Figure 8**).

## Discussion

Successful patient treatment benefits from early diagnosis and timely treatment; however, there are still some problems that need to be addressed in the early diagnosis and anti-tumor treatment of patients as well as in the diagnosis and treatment of CIP.

In the current case report, the patient was first diagnosed with NHL in the fifth month of treatment for lung squamous cell carcinoma according to SEER (Surveillance, Epidemiology, and End Results) recommendation (11). The patient had multiple metachronous primary tumors. The oncologist chose penpulimab for the treatment of lung cancer. This drug was approved for the first time in China in August 2021. It is a new PD-1 monoclonal antibody that is based on the IgG1 subtype. By binding to PD-1, it activates the immune surveillance and killing effect of T cells on tumors, inhibiting Fcγ receptor binding and Fc-mediated effector function, thereby reducing T cell apoptosis and clearance. Compared with other PD-1 inhibitors, as penpulimab and PD-1 dissociate more slowly, they have higher receptor occupancy and better cell activity and are recommended for Hodgkin’s lymphoma and non-small cell lung cancer (12). After assessing the patient’s medical condition, oncologists conducted a comprehensive review of the relevant literature and found some new evidence in the results of clinical trials (12, 13). The results of these studies indicated that the combination of penpulimab with chemotherapy consistently yielded favorable outcomes across all efficacy measures, significantly mitigating the risk of disease progression and mortality. Subsequently, in October 2021, the oncologist communicated with the patient and started treatment with penpulimab after obtaining informed consent. However, in our patient, lymph node enlargement occurred after five courses of drug treatment, and a lymph node biopsy was performed to diagnose NHL. Penpulimab has been recommended as a second-line treatment for Hodgkin’s lymphoma, but there have been no clinical trials for NHL. The occurrence of lymphoma during the use of a PD-1 inhibitor may be related to the fact that the patient’s lymphoma was a highly invasive NHL as well as to some genetic features such as chromosome 9p24.1 changes and PD-L1 expression. Early studies have found that the effect of PD-1 inhibitors in NHL treatment may not be ideal (14). The probability of having multiple primary cancers in patients with lung cancer is 21% (15), and the first metachronous multiple primary cancers of lung cancer rank ninth among metachronous multiple primary cancers, accounting for 3.2% (16). The cause of lymphoma in this patient may have been related to the following factors: 1) There may be a common carcinogenic mechanism for the development of lymphoma and lung cancer (17); 2) Tobacco exposure increases the incidence of multiple primary cancers (18), especially diffuse large B-cell lymphoma (19); 3) Occupational and environmental pesticide exposure: the patient was a farmer. Several studies have shown that occupational agricultural exposure is associated with a high incidence of NHL (20, 21) because exposure to agricultural pesticides increases the risk of diffuse large B-cell lymphoma (22); 4) Role of chemotherapeutic drugs: chemotherapeutic drugs can cause DNA strand breaks, cell transformation, mutations, and chromosomal aberrations, and can be anticancer and carcinogenic. Several classes of chemotherapeutic agents are associated with treatment-related tumors, including alkylating agents, topoisomerase II inhibitors, and antimetabolites (23); 5) Immunosuppression: patients experience bone marrow suppression and low immune function after chemotherapy, which diminishes the surveillance and killing effect on cancer cells. Some studies have reported that immunosuppression is the most common risk factor for NHL (24). Duration of Penpulimab: although the mean elimination half-life of penpulimab is 23.3 d, anti-PD-1 therapy generates polyclonal and memory-adaptive antitumor immunity that can control disease heterogeneity and reset tumor-host immune interactions to cancer rejection (25, 26). The Gustave-Roussy Cancer Center in France published the results of a large retrospective study in which 39 patients were forced to discontinue anti-PD-1 drugs early after treatment initiation for less than 2 years for several reasons. Of these patients, 15 experienced disease relapse, with a median time from discontinuation to relapse of 4 months, whereas remission continued in the remaining 24 patients. Comparing the 15 patients who relapsed with the 24 patients who did not relapse revealed that the duration of PD-1/PD-L1 monoclonal antibody was longer before discontinuation in the patients who did not relapse (15.5 months vs. 11 months) (27). Therefore, anti-PD-1 drugs have a “long tail effect,” which is why lung tumors do not recur immediately after patients stop taking the drugs, but they eventually regrow.

CIP epidemiology and risk factors: The incidence of CIP is 3–5%, but the mortality rate is 10–17%, accounting for 35% of deaths associated with PD-1 and PD-L1 inhibitors (28, 29). CIP occurs over a large period, ranging from 0.3 to 19.2 months of ICI treatment, with a median time of 2.8 months (30). Our patient developed a lung injury approximately 6 months after the start of penpulimab treatment (1 week after the last treatment), which was consistent with the time window reported in the literature. Studies have shown that prior lung disease, tumor type, use of PD-1 inhibitors, combination therapy, chest radiotherapy, smoking history, and age >70 years are risk factors for CIP (31, 32), The main risk factors for our patient were lung squamous cell carcinoma, a smoking history, and the use of PD-1 inhibitors. In addition, the patient was treated with a combination of rituximab therapy before the onset of B-cell depletion disrupted the T helper (Th)17/regulatory T cell (Treg) balance, with a decrease in Tregs and an increase in Th17, leading to complement activation, cytokine release (especially IL-6), granulocyte and monocyte recruitment, and immune hyperactivation (33). This action is similar to the mechanism by which PD-1 leads to CIP (34, 35). Therefore, the combination of two drugs will increase the antitumor effect and may simultaneously aggravate the immune imbalance of patients, thus leading to the development of CIP.

Clinical manifestations of CIP include dyspnea (53%), cough (35%), fever (12%), and chest pain (7%), with a rapid onset of respiratory failure in severe cases (36). Patient presentation often varies widely, with the literature reporting that popping sounds in the lungs can occasionally be heard on auscultation in patients with grade 3 (37), whereas one-third of patients may not have corresponding symptoms at presentation (30). Therefore, it is impossible to distinguish CIP from other lung diseases based on clinical presentation. The patient begins with dyspnea with persistent high fever, which may be related to the release of cytokines caused by ICI-induced T-cell attack on self-antigens (1). Clinical manifestations are easily confused with pulmonary infection; therefore, it is important to understand the medical history for diagnosis. During the management of critically ill patients, intensive care physicians often focus on the acute pathophysiological changes in the patient and neglect to review the medical history. A review of prior treatment for oncology patients may be an important aspect for intensive care physicians.

There are no specific imaging changes in CIP, although some studies have shown that the radiology of CIP mostly shows ground glass, solid, and grid shadows, most commonly in the subpleural and basal regions of both lungs (5). CT can detect CIP early when it is in a reversible stage and help identify other etiologies that can explain the patient’s symptoms (38). However, patients with different underlying conditions may also have different imaging findings. In patients treated with radiotherapy, the first sites of CIP are mostly located within the radiation field area (39); In patients with non-small cell lung cancer, CIP is typically characterized by a non-segmental distribution of ground glass and solid shadows in the dominant lung or bilaterally opposite the tumor in 54.55% and 31.82% of cases, respectively (28, 40); therefore, knowledge of the patient’s underlying disease may be further verified by imaging. The common imaging patterns of CIP are organized pneumonia, non-specific interstitial pneumonia, allergic pneumonia, acute interstitial pneumonia (AIP) (41), and bilateral pleural effusion in severe AIP (42), of which, AIP is the most serious. The imaging pattern in our patient was AIP, which showed diffuse ground glass shadow, grid shadow, and patchy solid lesions in both lungs with various manifestations, such as distended bronchial dilatation and pleural effusion, involving most of the lungs, mainly in both lower lungs.

Patients with CIP often have severe hypoxemia and require further respiratory support during the acute phase. Current guidelines do not specify the mode of support for this group of patients, and evidence suggests that mortality rates for intubation and invasive mechanical ventilation in immunodeficient patients are as high as 50–70% (43, 44) Pooled analysis showed that the use of non-invasive ventilation reduced mortality, the need for intubation, and the incidence of in-hospital pneumonia in this population. The expected effect of non-invasive ventilation in immunocompromised patients with acute respiratory failure outweighs adverse outcomes in most cases (45). Therefore, we chose noninvasive mechanical ventilation as the initial respiratory support strategy. A large body of clinical evidence suggests that prone position ventilation is sufficient to improve the prognosis of patients with ARDS (46), whereas the prone position improves oxygenation in patients with acute hypoxic respiratory failure without tracheal intubation (47). The awake-prone position significantly reduces intubation rates in patients with ARDS requiring high-flow oxygenation and noninvasive positive-pressure ventilation (48). The AIP imaging manifestation of our patient was characterized by rapidly progressed hypoxemia. In the early exudative phase of AIP, high-resolution CT showed bilateral patchy ground-glass shadows, often accompanied by gravity-dependent pulmonary consolidation (49). Biopsy shows diffuse alveolar damage in the form of AIP tissue, which is not significantly different from the histological pattern of ARDS (50). Therefore, AIP-type pneumonia is also called ARDS (6). According to the treatment strategy for ARDS, we administered small doses of sedative and analgesic drugs during non-invasive ventilation and prone position ventilation. After the above respiratory support, the hypoxia of the patients was significantly improved, indicating that awake prone position ventilation can improve the clinical symptoms of patients with CIP. However, currently, there is still a lack of research on the pathophysiological changes in patients with CIP after prone position ventilation. More research is needed to determine the clinical benefits of the prone position in patients with CIP and hypoxemia.

mNGS also played an important role in the diagnosis and treatment of this patient. By sequencing the DNA/RNA of clinical samples, mNGS can monitor a variety of pathogenic microorganisms without bias. Negative results have a good negative predictive value for excluding infections. Zhang et al. showed that the negative predictive value of mNGS was high (79.4%) and could be used to screen febrile patients for infection to reduce the overuse of antibiotics and the number of days of antibiotic treatment (51). Qian et al. analyzed 1021 patients who underwent mNGS testing and found that the negative predictive value of mNGS could be as high as 97.89%, and noted that when the mNGS result was negative, the negative prediction accuracy of the original sample was significant (52). Therefore, mNGS can assist in making rapid decisions when the etiology of a patient is unclear and it is particularly difficult to distinguish between infectious and non-infectious diseases. CIP can co-exist with infectious pneumonia (53). mNGS can guide precise antibiotic therapy for CIP co-infections. Our patient had low cellular immunity, and the administration of high-dose hormone therapy on this basis may have increased the patient’s risk of infection, which is often the result of a combination of viral, bacterial, and fungal infections in such patients (54). Empirical anti-infective regimens are often broad-spectrum antibiotics combined with antifungal or antiviral therapy. In contrast, the negative alveolar lavage fluid mNGS result in this patient provided evidence to rule out infection, rapidly discontinue antibiotics, and avoid overexposure to antibiotics. There are no reports on the value of mNGS for diagnosis in CIP-related literature. The success of our diagnostic treatment was partly due to the negative result of this test, but the interpretation of its background bacteria may vary in different populations; therefore, the value of this test in the diagnosis of CIP still needs to be validated by more clinical data.

## Conclusion

We report a case of severe CIP caused by a novel PD-1 inhibitor, penpulimab. Clinically, chest CT should be performed as early as possible for those who develop respiratory symptoms after ICI administration, and the possibility of CIP should be considered. CIP needs to be differentiated from infection, and bronchoalveolar lavage mNGS examination can rapidly and accurately screen for infection and guide the use of antimicrobial drugs. Early recognition and timely intervention are key to successful CIP treatment. As opposed to the concept, “To a man with a hammer, everything looks like a nail,” thinking outside the box and focusing on critical information that can easily be overlooked may be the cornerstone of successful treatment.

## Data availability statement

The original contributions presented in the study are included in the article/supplementary material. Further inquiries can be directed to the corresponding authors.

## Ethics statement

The studies involving human participants were reviewed and approved by Sichuan Academy of Medical Sciences and Sichuan Provincial People’s Hospital. The patients/participants provided their written informed consent to participate in this study. Written informed consent was obtained from the individual(s) for the publication of any potentially identifiable images or data included in this article.

## Author contributions

RL and RG treated the patient. RG and FY wrote the case report. RL and XL mainly revised this report. All authors made substantial contributions to data interpretation, discussion, manuscript preparation, review and revision. All authors read and approved the final manuscript.

## Glossary

**Table:**

CIP	checkpoint inhibitor pneumonitis
WHO	World Health Organization
ICI	immunecheckpoint inhibitors
IgG1	immunoglobulin G1
BMI	body mass index
GM-TEST	Glactomannan Test TEST (1 3) - b -D dextran test
PCP	pneumocystis carnii pneumonia
TORCH	toxoplasma rubella virus cytomegalovirus herpes simplex virus
AP	albumin paclitaxel+lobaplatin
CHOP	rituximab+cyclophosphamide+adriamycin+vincristine+prednisoneIv intravenous injection
Medr	methylprednisolone
Po	oral administration
PET-CT	Positron Emission Tomography-Computed Tomography
ICU	Intensive Care Unit
FDG	fluorodeoxyglucose
NHL	non-Hodgkin’s lymphoma
mNGS	metagenomic next-generation sequencing
EMSO	European Society for Medical Oncology
NIV	noninvasive ventilation
SEER	Surveillance Epidemiology and End Results
ADCC	antibody-depenadent cell-mediated cytotoxicity
ADCP	Antibody-dependent cell-mediated phagocytosis
HL	Hodgkin’s lymphoma
DLBCL	especially diffuse large B-cell lymphoma
NSIP	non-specific interstitial pneumonia
HP	allergic pneumonia
AIP	acute interstitial pneumonia
NIV	noninvasive ventilation
ARF	acute respiratory failure
ARDS	Acute Respiratory Distress Syndrome
HRCT	highresolution computed tomography
DAD	diffuse alveolar damage
NPV	negative predictive value
